# Oral Pharmacokinetics of a Chitosan-Based Nano- Drug Delivery System of Interferon Alpha

**DOI:** 10.3390/polym11111862

**Published:** 2019-11-11

**Authors:** Julieta C. Imperiale, Inbar Schlachet, Marianela Lewicki, Alejandro Sosnik, Mirna M. Biglione

**Affiliations:** 1 Instituto de Investigaciones Farmacológicas (ININFA), CONICET-Universidad de Buenos Aires, Buenos Aires C1113AAD, Argentina; julietaimperiale@gmail.com; 2 Laboratory of Pharmaceutical Nanomaterials Science, Department of Materials Science and Engineering, Technion-Israel Institute of Technology, Technion City, Haifa 3200003, Israel; inbarschlachet@gmail.com; 3 Instituto de Investigaciones en Microbiología y Parasitología Médica (IMPAM), CONICET-Universidad de Buenos Aires, Buenos Aires C1121ABG, Argentina; vetmarianelalewicki@gmail.com; 4 Instituto de Investigaciones Biomédicas en Retrovirus y Sida (INBIRS), CONICET-Universidad de Buenos Aires, Buenos Aires C1121ABG, Argentina

**Keywords:** IFNα, polymeric nanoparticles, oral protein delivery, in vitro intestinal permeability, oral pharmacokinetics

## Abstract

Interferon alpha (IFNα) is a protein drug used to treat viral infections and cancer diseases. Due to its poor stability in the gastrointestinal tract, only parenteral administration ensures bioavailability, which is associated with severe side effects. We hypothesized that the nanoencapsulation of IFNα within nanoparticles of the mucoadhesive polysaccharide chitosan would improve the oral bioavailability of this drug. In this work, we produced IFNα-loaded chitosan nanoparticles by the ionotropic gelation method. Their hydrodynamic diameter, polydispersity index and concentration were characterized by dynamic light scattering and nanoparticle tracking analysis. After confirming their good cell compatibility in Caco-2 and WISH cells, the permeability of unmodified and poly(ethylene glycol) (PEG)-modified (PEGylated) nanoparticles was measured in monoculture (Caco-2) and co-culture (Caco-2/HT29-MTX) cell monolayers. Results indicated that the nanoparticles cross the intestinal epithelium mainly by the paracellular route. Finally, the study of the oral pharmacokinetics of nanoencapsulated IFNα in BalbC mice revealed two maxima and area-under-the-curve of 56.9 pg*h/mL.

## 1. Introduction

Biological drugs are increasingly positioned in the pharmaceutical market because their high affinity for the different therapeutic targets enhances treatment efficacy and lowers side-effects. However, their high molecular weight, low lipophilicity and polyelectrolyte nature preclude absorption by transmucosal routes that are more patient-compliant. Besides, in the case of oral administration, they are exposed to hydrolytic and enzymatic degradation conditions along the gastrointestinal tract (GIT) that compromise their oral bioavailability [1].

Interferon alpha (IFNα) is a cytokine that exhibits a broad spectrum of antiviral, immunomodulatory, antiproliferative and antitumoral activities [2]. IFNα stimulates the innate-immune response and directs the transition from innate to acquired immunity and induces the differentiation of monocytes into dendritic cells [3], which are antigen-presenting cells. In addition, the antitumor and antiviral activity of IFNα stems from the activation of CD4 and CD8 T lymphocytes and natural killer cells, by increasing their cytotoxic activity and their ability to produce IFN gamma (IFNγ) that in turn enhances the secretion of other cytokines [4,5]. Furthermore, IFNα upregulates the expression of tumor-associated surface antigens and major histocompatibility complex (MHC) class I antigens that improve antigen recognition, induces pro-apoptotic genes and proteins, suppress anti-apoptotic genes, modulates cell-differentiation, and inhibits angiogenesis that is a key stage in tumor growth and metastasis [3]. All these mechanisms contribute to its activity against malignant cells. IFNα has been approved in the treatment of different types of cancer, including hairy cell leukemia, malignant melanoma, acquired immunodeficiency syndrome-related Kaposi’s sarcoma, follicular non-Hodgkin’s lymphoma and condyloma acuminate [6], and chronic viral infections such as hepatitis B and C [6]. To treat these diseases, IFNα is daily administered by subcutaneous or intramuscular injection, which is associated with strong pain and poor patient compliance. In addition, the half-life of IFNα is very short, e.g., 2.2 and 2.9 h after intramuscular and subcutaneous administration, respectively [7]. Commercial formulations of poly(ethylene glycol) (PEG)-modified (PEGylated) IFNα (e.g., Pegasys^®^ and PegIntron^®^) prolong the half-life of IFNα in the systemic circulation enabling weekly administration, although it is associated with a significant activity loss of 80% with respect to the non-PEGylated form [8]. In this framework, the investigation of new IFNα formulations with a better benefit–risk ratio is called for. Polymeric-based nanoparticulate systems are able to load therapeutic proteins maintaining their conformational structure and stability and, thus, retaining their biological activity [9,10]. Furthermore, polymeric carriers protect the cargo from chemical and/or enzymatic degradation [9]. They can effectively cross the intestinal barrier depending on the physicochemical properties of the polymer (e.g., hydrophilicity/hydrophobicity, functional groups) and the size, shape, surface area and charge of the carrier [9]. Most studies in the field of polymeric-based particulate systems for oral delivery of therapeutic proteins have been focused on the encapsulation of insulin [11,12,13,14]. Due to the promising results obtained, the advance towards other proteins is of interest. In this context, previously we developed recombinant IFNα-loaded chitosan (CT) nanoparticles (IFN-CT-NPs) to improve the stability and the absorption of the drug in the gastrointestinal tract. Remarkably, this carrier does not jeopardize the biological activity of the protein and enables its oral absorption in CF1 mice [15]. However, aiming to confirm whether nanoencapsulation improves gastrointestinal absorption or not, in this preliminary study, the oral pharmacokinetics was assessed only at one single time point, which is insufficient to plan preclinical trials in diseased animal models [15]. The assessment of the complete pharmacokinetic profile is needed to establish a possible administration regimen and pave the way for the bench-to-bedside translation.

In this work, we produced IFN-CT-NPs by the ionotropic gelation method and fully characterized the nanoparticle particle hydrodynamic diameter (D*_h_*), size distribution (expressed as polydispersity index, PDI) and concentration by dynamic light-scattering (DLS) and nanoparticle-tracking analysis (NTA). Then, the cell compatibility of the nanoparticles was assessed in Caco-2 (a model of intestinal epithelium) and WISH cells (a human amnion-derived cell line) and the permeability of unmodified and PEGylated nanoparticles was measured in monoculture (Caco-2) and co-culture (Caco-2/HT29-MTX) cell monolayers; HT29-MTX is a mucin-secreting cell line. Finally, the complete oral pharmacokinetics curve of nanoencapsulated IFNα was measured for the first time in BalbC mice. Overall results highlight the promise of this novel nanoformulation for the safe and effective oral administration of this key biological drug.

## 2. Materials and Methods 

### 2.1. Materials

CT (GC9009, batch 769FGD, molecular weight of ~50,000 g/mol, degree of deacetylation of ~94% and viscosity of ≤100 mPa.s) was purchased from Glentham Life Sciences (Corsham, UK). The degree of deacetylation measured in our laboratory by proton nuclear magnetic resonance (1H-NMR, 400-MHz Bruker^®^ Avance III High Resolution spectrometer, Bruker BioSpin GmbH, Rheinstetten, Germany with SpinWorks 4.0 software, University of Manitoba, Winnipeg, MB, Canada) and using 5% *w*/*v* deuterium oxide (D_2_O, Sigma-Aldrich, St. Louis, MO, USA) solution and trifluoroacetic acid (5% *v*/*v* in D_2_O, Sigma-Aldrich) was 94.5% [16,17]. The number- and weight-molecular weight of CT were 53,000 and 79,000 g/mol, respectively, as determined by gel permeation chromatography [17]. Sodium tripolyphosphate pentabasic (TPP) was supplied by Sigma-Aldrich (USA). Acetic acid was purchased from Merck Chemicals GmbH (Darmstadt, Germany) and IFNα-2b (BIOFERON^®^, lyophilized powder) from BioSidus (Buenos Aires, Argentina). PEG-carboxymethyl (mPEG5000-COOH, molecular weight of 5000 g/mol) was supplied by Laysan Bio, Inc. (Arab, AL, USA).

### 2.2. Preparation of Interferon Alpha (IFNα)-Loaded Nanoparticles

Blank and IFNα-loaded CT-NPs were prepared by the ionotropic gelation method between the polycationic CT and TPP anions. Briefly, TPP (1.8 mL, 1 mg/mL in water) was added dropwise to a CT solution (4.5 mL, 2 mg/mL) that contained IFNα [24 μg, equivalent to 5 milli-international units (MIU)/batch] using a 21G1 1/2 needle (internal diameter = 0.80 mm, length = 38 mm) and infusion pump (flow of 15 mL/h, PC11U, APEMA S.R.L., Buenos Aires, Argentina) under magnetic stirring, at room temperature [15]. The nanosuspension was magnetically stirred (15 min) at room temperature to consolidate the nanoparticles. NPs with conserved protein bioactivity and slow release at low pH (stomach) and faster release at neutral pH (small intestine) were obtained, as reported elsewhere [15]. To assess the effect of PEGylation on the permeability of CT-NPs, we prepared PEG.CT-NPs by using a mixture of pristine CT and PEGylated CT (PEG.CT) in a 1:1 weight ratio. CT was PEGylated by a two-step covalent coupling protocol reported by Chang et al. [18]. Briefly, mPEG5000-COOH (50 mg) was added to a solution of N-hydroxy succinimide (288 mg, NHS, 5 mM, Thermo Fisher Scientific, Waltham, MA, USA), 1-ethyl-3-(3-dimethylaminopropyl)carbodiimide hydrochloride (191.7 mg, EDC, Thermo Fisher Scientific) in 2-(N-morpholino) ethanesulfonic acid buffer (MES, 0.05M, 5 mL, Molekula, Irvine, CA, USA). The solution was kept under constant magnetic stirring at room temperature for 15 min. Then, activated mPEG5000-COOH was reacted with the primary amine moieties of CT in solution (2 mg/mL, 125 mL) dissolved in acetic acid (0.33% *v*/*v*). The product was dialyzed for 72 h and lyophilized (Labconco Free Zone 4.5 plus L Benchtop Freeze Dry System, Kansas City, MO, USA). PEG.CT was characterized by Fourier transform-infrared spectroscopy (FTIR) using an Equinox 55 spectrometer (Bruker Optics Inc., Ettlingen, Germany) and KBr disks (Merck KGaA, Gernsheim, Germany). The scanning range was 4000 to 400 cm^−1^, 32–64 scans and a resolution of 4 cm^−1^. In addition, the PEGylated product was analyzed by proton-nuclear magnetic resonance spectroscopy (^1^H-NMR, 400 MHz Bruker^®^ Avance III High Resolution Spectrometer, Ettlingen, Germany) using dimethyl sulfoxide-*d6* (DMSO-*d6*, Sigma-Aldrich) as solvent. Chemical shifts are reported in ppm using the peak of DMSO at 2.50 ppm as internal standard. The weight content of mPEG5000 in CT was estimated by interpolation in a calibration curve built from physical mixtures of CT and mPEG5000-COOH in acetic acid-*d4* using different CT:mPEG weight ratios between 0.02 and 0.7. Characteristic signals of each compound in the physical mixtures, namely 2.8 ppm (HC-NH_2_) of CT and 3.3 ppm (CH_3_O) of mPEG5000-COOH were used for the integration (*R*^2^ = 0.9766).

### 2.3. Characterization of the Nanoparticles

#### 2.3.1. Hydrodynamic Diameter, Polydispersity Index and Zeta-Potential 

The D*_h_* and PDI of fresh CT-NPs, PEG.CT-NPs and IFN-CT-NPs were measured by DLS (Zetasizer Nano-ZS, Malvern Instruments, Worcestershire, UK) equipped with a He–Ne (633 nm) laser and a digital correlator ZEN3600 using an angle of *θ* = 173° to the incident beam, at 25 °C. Results are expressed as number-based distribution of three samples prepared under identical conditions and each one of them is the result of at least four runs. The zeta-potential (Z-potential) of freshly prepared CT-NPs and IFN-CT-NPs was measured with the same equipment (pH = 5.5), at 25 °C.

#### 2.3.2. Nanoparticle-Tracking Analysis 

The quantification of the NP concentration (particles per mL of suspension) and the visualization of their Brownian motion was carried out by NTA (NanoSight^®^ NS500-Zeta HSB system with a high sensitivity camera and 638 nm laser for fluorescence analysis, Malvern Instruments, Malvern, UK) under scattering mode. 

#### 2.3.3. Stability of Nanoparticles as a Function of pH

Fresh NPs were diluted in media of pH 1 and 4 and incubated for 4 h and of pH 6, 7, 7.4 and 8 and incubated for 6 h (1:6 volume ratio of NPs:medium), at 25 and 37 °C. Then, the D*_h_* and the Z-potential were measured by DLS, as was described above. 

#### 2.3.4. Drug-Encapsulation Efficiency 

The encapsulation efficiency (*%EE*) of IFNα was determined by an indirect method [15]. Briefly, the amount of free drug in a sample of three independent batches of CT-NPs produced under identical conditions was measured with a commercial enzyme-linked immunosorbent assay kit (ELISA, Affimetrix, eBiosciences, USA). The *%EE* was calculated according to Equation (1).

*%EE* = [(*D_o_* – *D_f_*)/*D_o_*] × 100 (1)

where *D_o_* is the total amount of IFNα used in the preparation of the NPs and *D_f_* is the amount of free IFNα quantified in the supernatant after the encapsulation process.

The drug loading (*%DL*) was estimated according to Equation (2).

*%DL* = (IFNα_NP_/NP_t_) × 100 (2)

where IFNα_NP_ is the weight of IFNα used in the production of the NPs (24 μg) and NP_t_ is the total weight of NPs produced in one batch (~9 mg).

### 2.4. Cell Studies In Vitro

#### 2.4.1. Cells 

The human epithelial adherent cell line WISH was kindly donated by Prof. José Luis López from the Department of Virology (Faculty of Pharmacy and Biochemistry, University of Buenos Aires, Argentina). Cells were cultured in Eagle’s Minimum Essential Medium (EMEM, Life Technologies Corp., USA) supplemented with 10% heat-inactivated fetal bovine serum (Internegocios, Mercedes, Argentina), penicillin, L-glutamine and no essential amino acids (Sigma-Aldrich). Cells were maintained at 37 °C in humidified air with 5% of CO_2_. Cells were collected every 3-4 days (TrypLE Express enzyme, Gibco, Waltham, MA, USA) and the number of living cells were quantified by the trypan blue exclusion assay 0.4% (Sigma-Aldrich). The human epithelial cell line Caco-2 (HTB-37TM) was supplied by ATCC^®^ (Manassas, VA, USA) and the mucin-secreting HT29-MTX cell line was purchased from Sigma-Aldrich. These cells were cultured in Dulbecco’s Modified Eagle’s Medium (DMEM, Life Technologies Corp.) supplemented with l-glutamine, 10% heat-inactivated fetal bovine serum (FBS, Sigma-Aldrich) and penicillin/streptomycin (5 mL of a commercial mixture of 100 U per mL penicillin + 100 μg per mL streptomycin per 500 mL medium, Sigma-Aldrich), maintained at 37 °C in a humidified 5% CO_2_ atmosphere and split every 4–5 days. 

#### 2.4.2. Cell Compatibility and Permeability Assays

The cell compatibility of CT-NPs and PEG.CT-NPs was evaluated in the Caco-2 cell line. For this, cells were cultured on 96-well plates at a density of 2.5 × 10^4^ cells/well for 24 h to reach confluence. Then, the cell medium was replaced by a mixture of fresh medium cell (100 μL) and a dilution (100 μL) of CT-NPs or PEG.CT-NPs in cell medium. All NPs were previously sterilized by filtration (MF-MilliporeTM Membrane Filter, 0.22 μm pore size, Merck KGaA). Cells were exposed to the NP suspensions for 24 h, the medium was removed, and new medium (100 μL) and sterile 3-(4,5-dimethylthiazol-2-yl)-2,5-diphenyltetrazolium bromide solution (MTT, 25 μL, 5 mg/mL, Sigma-Aldrich) was added. Cells were incubated for 4 h at 37 °C under 5% CO_2_ atmosphere, the medium was removed, formazan crystals dissolved with DMSO (Sigma-Aldrich), and then quantified spectrophotometrically at 530 nm with reference at 670 nm (Multiskan GO, Thermo Fisher Scientific Oy, Vantaa, Finland). Cells cultured in medium without NPs were used as control (100% viability). The cell compatibility of the NPs selected to be orally administered to mice was also assessed in WISH cells. For this, cells were seeded onto 96-well plates at a density of 2.5 × 10^4^ cells/well and allowed to grow for 24 h to reach confluence. Then, the cell medium was replaced by a mixture of fresh medium (100 μL) and a dilution (100 μL) of (i) blank CT-NPs, (ii) IFN-CT-NPs, (iii) CT solution and (iv) free IFNα solution in cell medium. All specimens were previously sterilized by filtration. Cells were exposed to each treatment for 24 h. Then, the supernatant was withdrawn and the commercial reactive CellTiter 96^®^ AQueous One Solution (Promega, Madison, WI, USA) containing 3-(4,5-dimethylthiazol-2-yl)-5-(3-carboxymethoxyphenyl)-2-(4-sulfophenyl)-2H-tetrazolium (MTS) was added. Cells were incubated for 20 min and the absorbance was measured at 492 nm using a microplate spectrophotometer (Multiskan GO). The percentage of viability was calculated by extrapolation from a calibration curve prepared with growing amounts of WISH cells (0.25, 0.5, 1.0, 1.5, 2.0 and 2.5 × 10^4^ cells/well) incubated only with culture medium. 

For permeability studies, in a monoculture model, Caco-2 cells (3 × 10^5^ cells per well) were seeded in cell culture inserts (ThinCert™, culture surface of 113.1 mm2, 3.0 μm pore size, Greiner Bio-One GmbH, Frickenhausen, Germany) maintained in 12-well plates (15.85 mm diameter, 16.25 mm height, Greiner CELLSTAR) with 0.5 and 1.5 mL of DMEM medium in the donor (apical) and acceptor (basolateral) chambers, respectively. Cells were incubated for 10–25 days, the culture medium was replaced every 2–3 days and the integrity of the cell monolayer was characterized by trans-epithelial electrical resistance (TEER) measurements performed with an epithelial volt-ohm-meter (EVOM2, World Precision Instruments, Sarasota, FL, USA). For permeability experiments, only inserts where the resistance was higher than 260 Ω cm^2^ were used. Permeability experiments were also performed in co-culture monolayers of Caco-2:HT29-MTX (9:1 number ratio). For this, the same cell density was seeded, and the protocol carried out as described above. The test sample consisted of a stock dispersion of blank CT-NPs and PEG.CT-NPs fluorescently-labeled by conjugation of fluorescein thioisocyanate (FITC, Sigma-Aldrich) to the amine groups of CT in the side chain and diluted with transport medium. This labeling pathway ensures that the label will not be released from the nanoparticles during the experiments. For preparation of FITC-labeled CT that was then mixed with PEG.CT to produce fluorescently-labeled PEGylated NPs, a synthetic method described elsewhere with a little modification was used [19]. Briefly, 20 mL of methanol was added to 20 mL of 1% *w*/*v* CT in a 0.05 M nitric acid solution. Then, 20 mL of a solution of FITC previously dissolved in methanol (2 mg/mL) was added under magnetic stirring. The reaction was allowed to proceed for 3 h at room temperature and protected from light. The resulting solution was purified by dialysis (nominal molecular weight cut-off of 3500 Da, Cellu Sup^®^ T1 nominal flat width of 46 mm, diameter of 29.3 mm, and volume/length ratio of 6.74 mL/cm; Membrane Filtration Products, Inc., Seguin, TX, USA) for 72 h and lyophilized. To produce FITC-labeled NPs, a mixture of unlabeled and fluorescently-labeled CT (weight ratio of 1:1) was used as described above. Immediately before the beginning of the experiment, the cell culture medium was replaced by Hank’s Balanced Salt Solution (transport medium, HBSS, Sigma-Aldrich) buffered to pH 6.8 with 25 mM of 4-(2-hydroxyethyl)-1-piperazineethanesulfonic acid (HEPES, Sigma-Aldrich) to a final concentration of 720 µg/mL in both the donor (apical, 0.45 mL) and the acceptor (basolateral, 1.2 mL) chambers. After 5, 10, 15, 30, 60, 90, 120, 180 and 240 min, 600 μL was extracted from each acceptor chamber to quantify the concentration of NPs that crossed the cell monolayer by fluorescence spectrophotometry (Fluoroskan Ascent Plate Reader, Thermo Fisher Scientific Oy) utilizing black 96-well flat bottom plates (Greiner Bio-One International GmbH, Kremsmünster, Austria) at wavelengths of 355 nm for excitation and 635 nm for emission. The apparent permeability coefficient (*P_app_*) was calculated according to Equation (3).

*P_app_* = *dc/dt* × 1/(A × C_o_) (3)

where *dc/dt* is the transport rate (μg.s^−1^) across the monolayer, Co is the initial concentration of NPs in the donor compartment (μg.cm^−3^), and A is the surface area of the membrane (cm^2^). 

#### 2.4.3. Cell Uptake

To gain further insight into the cell uptake of the NPs, the permeability test in the co-culture of Caco-2:HT29-MTX (9:1 number ratio) was continued for 24 h. Then, cells were harvested by trypsinization (trypsin-ethylenediaminetetraacetic acid 0.25%, Sigma-Aldrich), washed with fresh medium to remove non-internalized NPs and transferred to a 96-well plate with trypan blue (0.4% *w*/*v*, 50 μL, Sigma-Aldrich) to quench the fluorescence of the NPs adhered to the cell surface. As control of quenching, the fluorescence of a dilution of FITC-labeled NPs (without cells) with trypan blue was quantified. 

### 2.5. Oral Pharmacokinetics

The oral bioavailability was assessed in female BalbC mice aged 8–10 weeks (n = 20) purchased from the National University of La Plata (La Plata, Argentina). The preclinical protocol was approved by the Institutional Committee for the Care and Use of Experimental Animals (CICUAL) of the Faculty of Medicine (Resolution #2609/2016, University of Buenos Aires, Buenos Aires, Argentina). Animals were fasted for 2 h prior the administration. Then, IFN-CT-NPs (400 μL, 0.3 MIU) were orally administered by gavage using a straight stainless steel probe. The dose was selected based on the recommended dose for most therapies (5 MIU/m^2^) adjusted to the average body surface of each mouse. The value obtained was multiplied arbitrarily by 10 given that it is known that the oral bioavailability is lower than the parenteral one. Three samples were extracted from each mouse. The first two by sub-mandibular puncture, and the last one by cardiac puncture, after intraperitoneal administration of anesthesia (ketamine 150 mg/kg and xylazine 10 mg/kg, Sigma-Aldrich). Samples were obtained at the following time points post-administration: 15, 30, 45, 60, 120, 150, 180, 240, 300 and 360 min. As control, blood samples were obtained from mice (n = 2) by cardiac puncture at 30 min post-administration of the following treatments: (i) water, (ii) free IFNα, (iii) a mixture of IFNα and a CT solution and (iv) a mixture of free IFNα and blank CT-NPs. Samples were centrifuged to separate cells and plasma was frozen at −80 °C until analysis. Plasma IFNα levels were quantified by ELISA according the instructions of the manufacturer (Affimetrix, EBiosciences, Vienna, Austria). The following pharmacokinetic parameters were calculated using a non-compartmental model (TOPFIT program version 2.0, Dr. Karl Thomae Gmbh, Biberach, Germany): (i) maximum plasma concentration (C_max_), (ii) time to C_max_ (t_max_), (iii) the area-under-the-curve between 0 and ∞ (AUC_0-∞_) and the apparent half-life in plasma (*t_1/2_*).

### 2.6. Statistical Analysis

Statistical analysis was performed by *t*-test or one-way analysis of variance (ANOVA, significance level of 5%) with Bonferroni test using Graph Pad Prism version 7 for Windows (GraphPad, San Diego, CA, USA).

## 3. Results and Discussions

### 3.1. Synthesis and Characterization of PEGylated Chitosan

Aiming to compare the mucoadhesiveness/ mucopenetration of CT-NPs in a model of intestinal epithelium in vitro, we synthesized PEG.CT-NPs. This modification relies on the ability of PEG to minimize the interaction between CT and mucus [20]. For this, CT was primarily PEGylated by the condensation of the terminal carboxylate group of mPEG5000-COOH with the amine groups in the side-chain of CT utilizing the EDC/NHS chemistry [18]. The successful synthesis was confirmed by FTIR and ^1^H-NMR (Supplementary Materials, Figure S1). The FTIR spectrum showed the typical absorption bands of pristine CT at 3448 cm^−1^ corresponding to the overlapping of O–H and N–H stretching, at 2900 cm^−1^ due to CH_2_ groups, at 1670 cm^−1^ owing to the C=O stretching of the remaining amide, at 1617 cm^−1^ (weak band) due to N-H bending of amine and at 1087 cm^−1^ of C–O stretching of ether (Figure S1A). Conversely, the spectrum of PEG.CT showed the characteristic bands of CT together with an increase in the intensity of the peak at 1087 cm^−1^, which corresponds to the stretching vibrations of C–O–C bond, the main characteristic absorption band of PEG. Furthermore, a new strong band at 1560 cm^−1^ was consistent with the conjugation of PEG blocks to the CT side-chain through the formation of amide moieties. In addition, appearance of a new peak at 3.3 ppm in the ^1^H-NMR spectrum of PEG-CT confirmed the PEGylation (Figure S1B). This method was also utilized to quantify the PEG content for which a calibration curve CT/mPEG5000 physical mixtures with different weight ratios was built. The PEG content in PEG.CT was 12.6% *w*/*w*. 

### 3.2. Preparation and Characterization of the Nanoparticles

CT-NPs showed one single size population with D*_h_* of 47 nm and PDI of 0.47, as determined by DLS (Table 1). PEGylation led to a sharp D*_h_* growth to 93 nm (PDI = 0.32), due to the formation of a highly hydrated corona of PEG blocks (Table 1). In addition, the NP concentration (particles/mL) was quantified by NTA. Pristine (1.6 mg/mL) and PEGylated NPs (1.75 mg/mL) showed concentrations of 3 × 10^11^ ± 6 x 10^7^ and 3 × 1011 ± 3 × 10^10^, respectively (Table 1); the concentration of the NPs was adjusted to ensure identical CT concentration in both samples.

### 3.3. Cell Compatibility, Permeability and Uptake

**Figure 1:**
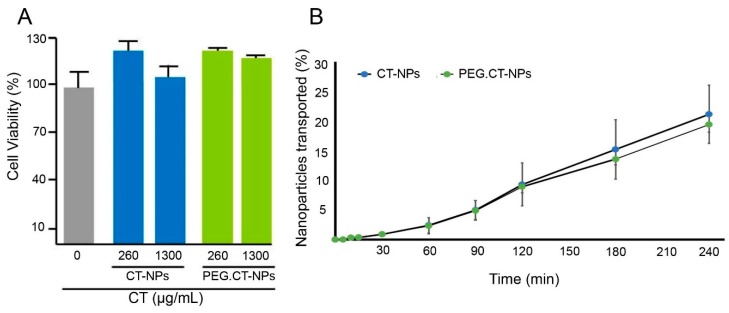
(**A**) Caco-2 cell viability upon exposure to different concentrations of CT-NPs and PEG.CT-NPs, as determined by the MTT (3-(4,5-dimethylthiazol-2-yl)-2,5-diphenyltetrazolium bromide solution) assay and (**B**) cumulative transport of CT-NPs and PEG.CT-NPs across Caco-2 cell monolayers. Values are expressed as the mean ± standard deviation (S.D.).

CT has good safety profile by the oral route, it is classified as a “generally recognized as safe” ingredient by the US Food and Drug Administration (FDA) and it has been approved as a food supplement in Japan, Italy, Finland and Brazil. CT is commercialized as ChitoClear^®^ by Primex to bind fatty acids and prevent their oral absorption [21]. In addition, the good cell compatibility of different types of CT-NPs, including amphiphilic ones, was reported elsewhere [22]. As a preamble to the permeability and uptake studies, the Caco-2 cell compatibility of CT-NPs and PEG.CT-NPs was assessed under the same conditions (time and concentration) used in the permeability assays. Both types of NPs showed approximately 100% viability (Figure 1A). Then, their permeability across a monolayer of Caco-2 cells was evaluated in order to learn whether PEGylation modifies the permeability or not. Caco-2 is a human epithelial cell line widely used as a model of the intestinal epithelial barrier [23]. Results showed that 19.4 ± 2.6% of PEG.CT-NPs and 21.1 ± 4.8% of CT-NPs crossed the Caco-2 monolayer after 4 h (Figure 1B). In addition, both curves displayed almost identical slopes and, as expected, similar *P_app_* values; 5.531 × 10^−6^ ± 6.504 × 10^−7^ and 6.064 × 10^−6^ ± 1.162 × 10^−6^ cm/s for PEGylated and unmodified NPs, respectively, with no statistically significant differences. These values suggest that these nanocarriers have moderate permeability [24]. 

**Figure 2:**
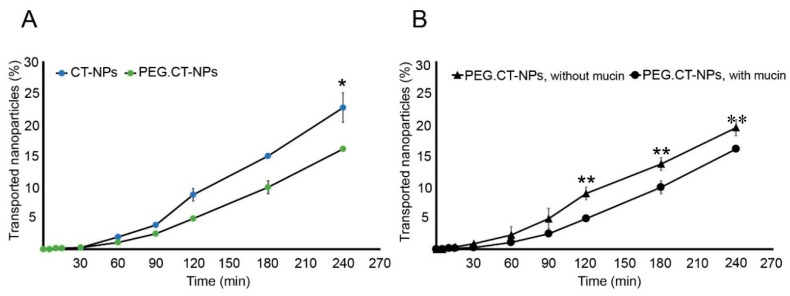
Cumulative transport of (**A**) CT-NPs and PEG.CT-NPs across a co-culture of Caco-2 cells:HT29 (9:1) and (**B**) PEG.CT-NPs across a monolayer of Caco-2 cells and a co-culture of Caco-2HT29 (9:1) cells. Values are expressed as the mean ± S.D. (n = 3). * Statistically significant difference in the transported nanoparticles (%) between non-PEGylated and PEGylated nanoparticles (*p* < 0.05); ** Statistically significant difference in the transport of PEGylated nanoparticles between cell monolayers without and with mucin (*p* < 0.05).

However, monoculture of Caco-2 monolayers is a simplified model of the intestinal epithelium because it lacks mucin. To more closely simulate the physiological conditions, a co-culture of Caco-2:HT29-MTX (9:1) cells was used. This model enabled to study the effect of PEGylation on the interaction of the NPs with mucus and the contribution of mucin to the permeability of the nanoparticles in a more preclinically relevant manner. Results showed that 16.0 ± 0.3% (*P_app_* = 4.486 × 10^−6^ ± 8.691 × 10^−8^ cm/s) and 22.6 ± 3.9% (*P_app_* = 7.006 × 10^−6^ ± 1.050 × 10^−6^ cm/s) of PEG.CT-NPs and CT-NPs, respectively, permeated the cell monolayer after 4 h (Figure 2A). These results indicated that the presence of mucin does not affect the permeability of pristine CT-NPs. Conversely, a significant decrease in the *P_app_* was observed for PEG.CT-NPs (*p* < 0.05) (Figure 2B). This behavior could be explained by two possible phenomena: (i) the formation of entanglements between PEG blocks and mucin that prevents NPs from reaching the apical surface of the epithelial cells; and (ii) a decrease in the concentration of the free amine groups of CT that are involved in the opening of epithelial tight junctions and the permeability of the nanoparticles by the paracellular pathway. Based on these results, CT-NPs were selected to conduct further permeability studies. 

Aiming to gain insight into the transport mechanisms involved in the permeability of CT-NPs across a co-culture Caco-2/HT29-MTX cell monolayer, the study was continued for 24 h. Findings confirmed that 41.0 ± 8.0% of the initial amount of nanoparticles permeated the monolayer detecting them in the acceptor chamber, while 45.7 ± 5.5% of the nanoparticles remained in the donor chamber. The difference to complete 100% of the initial amount of CT-NPs (13.3%) probably interacted with the cell monolayer by adsorption onto the cell surface or by cellular uptake [22]. To investigate if this percentage of nanoparticles was internalized or only adhered to cell surface, we trypsinized and washed the cells and quantified the fluorescence with and without the addition of trypan blue which is a dye that quenches external, though not internal fluorescence because it is unable to penetrate intact cell membranes. Before the addition of trypan blue, 7.6 ± 2.7% of CT-NPs were quantified from the cell suspension, a value that was quite similar to the theoretical value of 13.3%. Conversely, when the fluorescence was determined after the addition of trypan blue, values were undetectable. These results strongly suggested that CT-NPs are not endocytosed by enterocytes and that they were retained by the cell monolayer due to electrostatic interactions between the positively-charged CT and the negatively-charged cell membrane. Although the in vitro model used in this work does not rule out the transport of CT-NPs through M cells (not represented in the Caco-2/HT29-MTX co-culture model), our results are in line with transport by a paracellular transport. 

**Figure 3:**
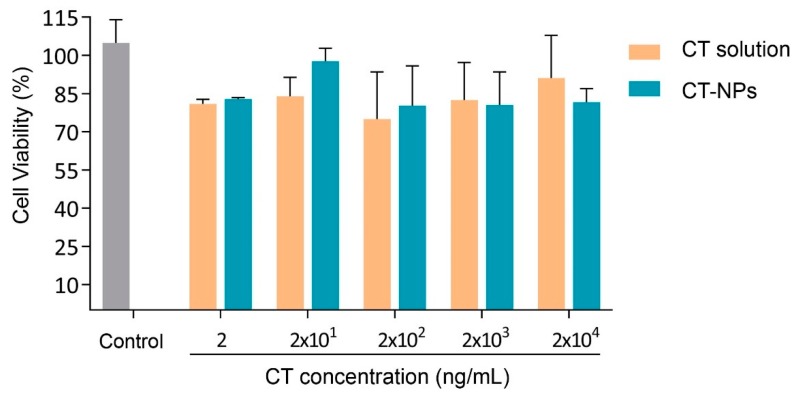
WISH cell viability upon exposure to different concentrations of CT solution and CT-NPs. Values are expressed as the mean ± S.D. (n = 3).

Finally, since the cell compatibility depends not only on the nanoparticle properties (e.g., composition and size) but also on the cell type, we also evaluated the compatibility of CT-NPs in the line of WISH human epithelial cells. Results showed that there were no significant differences in the viability of the WISH cells treated with CT-NPs (2–20,000 ng/mL CT) and the control (Figure 3). Equivalent doses of CT in solution did not show toxic effects. 

**Figure 4:**
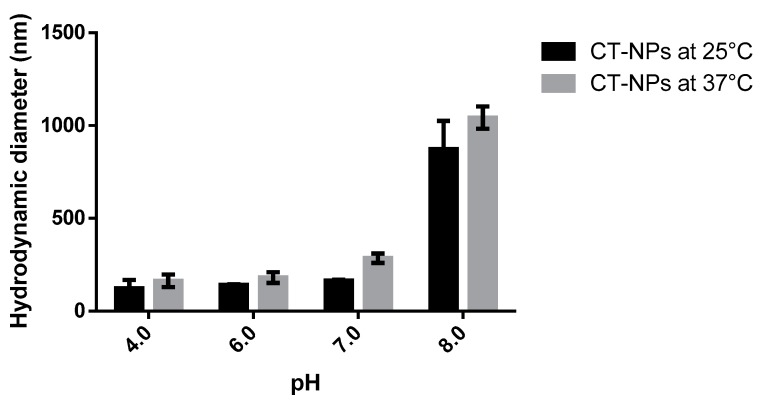
Stability of CT-NPs under different conditions of pH, at 25 and 37 °C.

The physical stability of CT-NPs was determined under physiologically relevant conditions of pH and temperature by measuring the D*_h_*. The gastric pH varies from 1–2 and 4–5, and the gastric emptying averages between 2 and 5 h [25]. Since the pH of fresh nanoparticles is approximately 5, we selected the harsher conditions with respect to pH and incubation time to challenge the physical stability of the delivery system. Accordingly, CT-NPs were incubated at pH 1 and 4 for 5 h, at 25 and 37 °C. Likewise, small intestinal emptying time averages between 3 and 6 h, while the pH increases from 6 to 7.4 in the terminal ileum [25]. Consequently, CT-NPs were also exposed to pH 6, 7, 7.4 and 8 for 6 h, at 25 and 37 °C. Results showed that CT-NPs incubated at pH 1, reduced their D*_h_* by half, determined as number-based distribution by DLS, at both 25 and 37 °C (Figure 4). Furthermore, a bimodal distribution profile was exhibited when the measurement was determined as intensity-based distribution by DLS, as opposed to the monomodal profile (one size population) of CT-NPs freshly prepared. These findings suggest that CT-NPs are unstable at pH 1. This can be explained because TPP is a polyprotic acid with four different p*K*_a_ values (p*K*_a,1_ = −∞, p*K*_a,2_ = 1.1, p*K*_a,3_ = 2.3, p*K*_a,4_ = 6.3, p*K*_a,5_ = 8.9). The low ionization of TPP at pH 1 weakens its binding capacity to the polycationic CT, thus resulting in the dissolution of the particle. On the other hand, the stability of CT-NPs increased at pH 4, 6 and 7. D*_h_* values of CT-NPs exposed to these media were similar among them at 25 °C despite the fact that p*K*_a_ of CT is 6.3. Notwithstanding, at 37 °C, the D*_h_* of CT-NPs exposed at pH 7 slightly increased. As expected, the higher the pH, the lower the Z-potential. The values obtained were ~+32, +8 and +4 mV at pH 4, 6 and 7, respectively, at both temperatures. At pH 8, there was a sharp increase of D*_h_*, with PDI > 0.700 due to the neutralization of CT which leads to a decrease of the electrostatic affinity between the amine groups of CT and TPP. This phenomenon could expect the aggregation of NPs. Accordingly, the Z-potential was neutral at both temperatures (0.06 and 0.01 mV at 25 and 37 °C, respectively).

The *%EE* of IFNα, as determined by ELISA, was 99.9% resulting in a high yield-production method. Loaded and unloaded CT-NPs exhibited a Z-potential greater than +30 mV. It must be stressed that adverse effects of IFNα are dose-dependent and it has been reported that low doses of IFNα may mimic natural defense processes and minimize adverse effects [26]. Since IFNα is a potent biological drug and the nanoencapsulation process increases its stability and protects it from degradation in the gastrointestinal tract, we encapsulated a low amount of biological drug (*%DL* = 0.27%) within the nanoparticles to study the complete oral pharmacokinetics profile. 

### 3.4. Oral Pharmacokinetics 

In a preliminary pharmacokinetic study, we showed that IFNα is detected in the systemic circulation 1 h after the oral administration of IFN-CT-NPs to CF1 mice [15]. Contrariwise, at this time point, the same dose of orally administered free IFNα could not be detected in plasma [15]. These results were in good agreement with studies conducted in mice, rabbits, dogs and monkeys [26]. Remarkably, our previous results were the first to show the oral absorption of this biological drug. However, these studies were conducted utilizing outbred mice that usually result in greater data variability and the plasma concentration was quantified at one single time point. To pave the way for a bench-to-bedside translation, a complete oral pharmacokinetic study is required. In this work, we used an inbred strain, female BalbC mice, to minimize variability. The main pharmacokinetic parameters are summarized in Table 2. 

**Figure 5:**
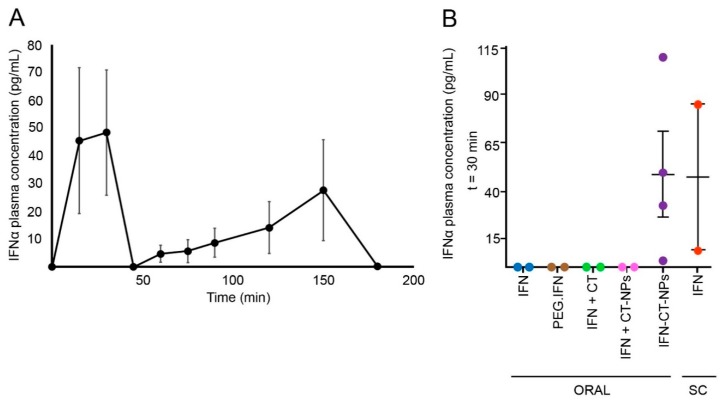
Oral pharmacokinetics of interferon alpha (IFNα). (**A**) Mean plasma concentration (± standard error of the mean (S.E.M.))—time profile of IFNα after oral administration of IFN-CT-NPs (dose = 0.3 MIU) to BALB/c mice (n = 20) and (**B**) IFNα plasma concentration (±S.E.M) in mice at 0.5 h after the oral administration of commercial free IFNα (IFN), commercial PEGylated-IFNα (PEG-IFN), a physical mixture of free IFNα and blank CT-NPs (IFN + CT-NPs), a physical mixture of free IFNα and a CT solution (IFN + CT) or IFN-CT NPs (n = 2 for all control groups). An additional group was subcutaneously treated with commercial free IFNα.

As shown in Figure 5A, after oral administration of a single dose of 0.3 MIU (1.4 pg) of IFN-CT-NPs, the concentration of IFNα in plasma showed two maxima peaks: the first at 0.5 h (C_max_ = 48.4 ± 22.5 pg/mL) and the second at 1.5 h (C_max_ = 27.6 ± 31.4 pg/mL). In addition, the levels of IFNα in plasma were undetectable 3 h after administration (limit of quantification 3 pg/mL). Two absorption peaks are typically associated with an enterohepatic recirculation phenomenon, although IFNα is a highly labile protein and recirculation is unlikely. In this context, the first peak would correspond to primary conventional intestinal absorption, while the second at a later time might be associated to lymphatic absorption that is slower than the intestinal one. It is important to stress that particles with sizes <3 μm can translocate to the gut-associated lymphoid tissue (GALT) migrating into the mesenteric lymph nodes and appearing in the lymph between 10 min and 3 h after administration, as a function of nutritional conditions and particle size [27]. Other possibility would involve the absorption of the drug in two different portions of the gut. The AUC_0_-∞ obtained was 56.9 pg*h/mL, which is a promising result considering that a pharmacokinetic study involving healthy volunteers showed that the C_max_ and AUC after the intravenous administration of IFNα-2b (5 MIU) were 853 pg/mL and 994 pg*h/mL, respectively, and 207.9 pg/mL and 2850 pg*h/mL, respectively, after the subcutaneous administration of the drug (5 MIU) [7]. Consequently, considering the dose adjustment, the values obtained in this study are in good agreement with those obtained in human volunteers. Similarly, the C_max_ and AUC obtained after a single subcutaneous injection of the drug (3 MIU) to patients with chronic hepatitis C virus infection were 42 pg/mL and 879 pg*h/mL, respectively [28]. 

As control, we confirmed that after the oral administration of free IFNα, PEGylated IFNα and free IFNα co-administered with either blank CT-NPs or a CT solution, IFNα could not detected at 0.5 h (the first maximum absorption time) (Figure 5B). Outstandingly, the concentration of IFNα detected in plasma after the subcutaneous administration of free IFNα (46.7 ± 38.3 pg/mL) was similar to that obtained with the oral administration of IFN-CT NPs (48.4 ± 22.5 pg/mL) after 2.5 h. These results highlight the potential of this nanocarrier for the improved oral delivery of this biological drug.

## 4. Conclusions

In this work, we produced CT-NPs and PEG.CT-NPs as a nanotechnology platform for the encapsulation and oral administration of IFNα. After the characterization of the nanoparticles, we studied their cell compatibility and permeability in vitro. These nanocarriers exhibited a moderate permeability across a co-culture Caco-2/HT29-MTX cell monolayer mainly by the paracellular pathway. PEGylation of the nanoparticles did not improve permeability and was detrimental most probably due to the formation of entanglements with mucin or the more limited ability of the nanoparticles to open epithelial tight junctions. Finally, we investigated for the first time the oral pharmacokinetic profile of this popular biological drug. Oral administration of IFN-CT-NPs led to a bioavailability of 56.9 pg*h/mL in BalbC mice. The concentration of IFNα detected in plasma was similar to the obtained by subcutaneous administration of free IFNα. These data highlight the potential of this nano-drug delivery system for the oral administration of this popular biological drug. Future studies will investigate the efficacy of this nanoformulation in animal models of diseases that are treated with IFNα as first-line medication.

## Figures and Tables

**Table 1:** Hydrodynamic diameter (D*_h_*), polydispersity index (PDI) and concentration of chitosan nanoparticles (CT-NPs) and PEG.CT-NPs, as measured by dynamic light scattering (DLS) and nanoparticle-tracking analysis (NTA).

Parameter	CT-NPs	PEG.CT-NPs
D*_h_* (nm)	47	93
PDI	0.47	0.32
Concentration (particles/mL)	3 × 10^11^ ± 6 × 10^7^	3 × 10^11^ ± 3 × 10^10^

**Table 2:** Pharmacokinetic parameters after the oral administration of a single dose (0.3 MIU, 1.4 pg, 0.07 pg/kg) of CT-NPs to BalbC mice.

Parameter	Value
*t*_max1_ (h)	0.5
*t*_max2_ (h)	1.5
*C*_max1_ (pg.mL^−1^)	48.4 ± 22.5
*C*_max2_ (pg.mL^−1^)	27.6 ± 31.4
*t*_1/2_ (h)	0.07 ± 0.02
AUC_0-∞_ (pg*h/mL)	56.92

## Supplementary Materials

The following are available online at https://www.mdpi.com/2073-4360/11/11/1862/s1: Figure S1. Characterization of PEG.CT. (A) Fourier transform infrared (FTIR) spectra of CT and PEG.CT and (B) proton nuclear magnetic resonance (1H-NMR) spectra of CT, mPEG5000, a physical mixture (PM) of mPEG and CT and PEG.CT.
