# Supplementary material for: Oral Pharmacokinetics of a Chitosan-Based Nano- Drug Delivery System of Interferon Alpha
Source: Polymers (Basel). 2019 Nov 11;11(11):1862. doi: 10.3390/polym11111862 (PMC6918283; doi:10.3390/polym11111862)
Supplement: Supplementary file 1 [file polymers-11-01862-s001.pdf]

Supplementary Materials:

Article

Oral Pharmacokinetics of a Chitosan-Based Nano-Drug Delivery System of Interferon Alpha

Julieta C. Imperiale [1], Inbar Schlachet [2], Marianela Lewicki [3], Alejandro Sosnik [2,*] and Mirna M. Biglione [4,*]

[1] Instituto de Investigaciones Farmacológicas (ININFA), CONICET-Universidad de Buenos Aires, Buenos Aires C1113AAD, Argentina; julietaimperiale@gmail.com

[2] Laboratory of Pharmaceutical Nanomaterials Science, Department of Materials Science and Engineering, Technion-Israel Institute of Technology, Technion City, Haifa 3200003, Israel; inbarschlachet@gmail.com

[3] Instituto de Investigaciones en Microbiología y Parasitología Médica (IMPAM), CONICET-Universidad de Buenos Aires, Buenos Aires C1121ABG, Argentina; vetmarianelalewicki@gmail.com

[4] Instituto de Investigaciones Biomédicas en Retrovirus y Sida (INBIRS), CONICET-Universidad de Buenos Aires, Buenos Aires C1121ABG, Argentina

* Correspondence: alesosnik@gmail.com or sosnik@technion.ac.il (A.S.); mbiglione@fmed.uba.ar (M.M. B.)

Received: 27 October 2019; Accepted: 7 November 2019; Published: date

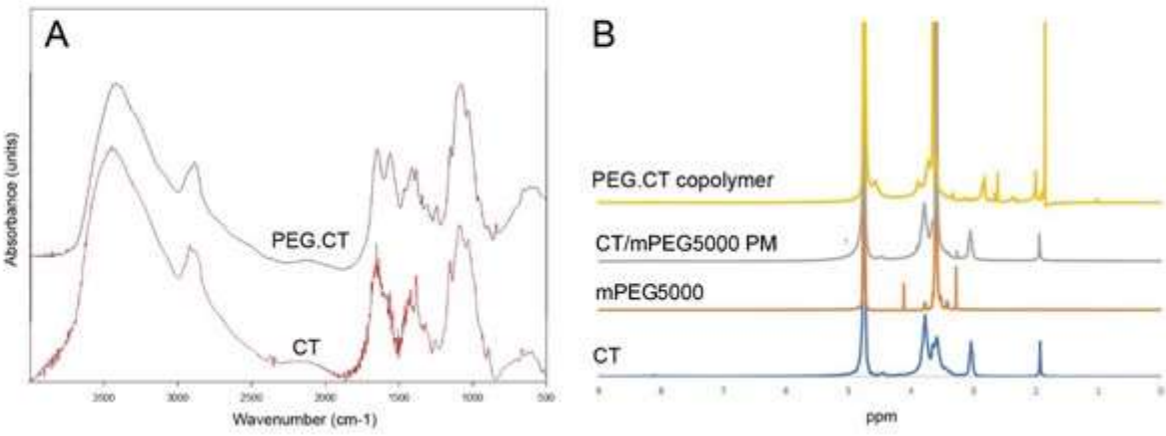

Figure S1. Characterization of PEG.CT. (A) FTIR spectra of CT and PEG.CT and (B) 1H-NMR spectra of CT, mPEG5000, a physical mixture (PM) of mPEG and CT and PEG.CT.
